# Complete circular assembly of *Histophilus somni* PDS1537697-81-1 genome and phenotypic characterization of its antibiotic susceptibility

**DOI:** 10.1128/mra.00170-24

**Published:** 2024-05-13

**Authors:** Anatoliy Trokhymchuk, Nathan Erickson, Cheryl L. Waldner, Matthew G. Links

**Affiliations:** 1 Department of Large Animal Clinical Sciences, Western College of Veterinary Medicine, University of Saskatchewan, Saskatoon, Saskatchewan, Canada; 2 Prairie Diagnostic Services Inc., Saskatoon, Saskatchewan, Canada; 3 Department of Animal and Poultry Science, College of Agriculture and Bioresources, University of Saskatchewan, Saskatoon, Saskatchewan, Canada; 4 Department of Computer Science, College of Arts and Science, University of Saskatchewan, Saskatoon, Saskatchewan, Canada; The University of Arizona, Tucson, Arizona, USA

**Keywords:** *Histophilus somni*, bacteria, genome, nanopore, MIC, minimum inhibitory concentration, antibiotic resistance

## Abstract

A *Histophilus somni* isolate from a clinically healthy, fall-placed calf was obtained upon arrival to a commercial feedlot. Fall-placed calves are commonly viewed to be at high risk for the development of bovine respiratory disease. The isolate was phenotyped for antimicrobial susceptibility and sequenced to obtain a complete, circular, genome assembly.

## ANNOUNCEMENT

*Histophilus somni* is a significant bacterial pathogen in the bovine respiratory disease (BRD) complex (1). Genomics-based tools can enhance diagnostic support for antimicrobial stewardship in BRD management but require the establishment of comprehensive reference data on isolates with known antibiotic susceptibility phenotypes (2).

In December 2015, a deep nasopharyngeal swab (Tiegland Modified Culture Swab, Jorgensen Laboratories, LLC, Loveland, CO, USA) was collected from a clinically healthy auction-sourced steer on arrival to a commercial feedlot (University of Saskatchewan Animal Research Ethics Board Certificate of Approval AUP#20150075), placed in Amies transport medium (eSwab Transport System Copan Diagnostics Inc., Murrieta, CA, USA), and delivered on ice to Prairie Diagnostic Services (PDS; Saskatoon, SK, Canada). *H. somni* PDS1537697-81-1 was isolated by plating 10 µL of the transport medium on a Chocolate agar plate, incubating for 48 h at 35°C in 5% CO_2_, and identified by MALDI-TOF MS (Microflex LT system, Bruker Daltonics, Billerica, MA, USA) as previously described (3). The isolate was cryopreserved, in Tryptic Soy Broth (TSB, Difco Laboratories, Detroit, MI, USA) containing 15% glycerol at −80°C, after one passage. Recovery from cryopreservation was done by quadrant inoculation of a Chocolate agar plate and 18-h incubation at 35°C in 5% CO_2_. The culture purity and the organism identity were re-confirmed by analyzing single colonies of a characteristic morphology on a MALDI-TOF MS.

Antimicrobial susceptibility testing, as a single diagnostic submission, was performed by broth microdilution method (ThermoFisher Scientific, Nepean, ON, Canada) using 96-well Sensititre microtiter plates (BOPO6F, ThermoFisher Scientific; Table 1) as described previously (4).

Bacterial DNA was extracted from a fresh Chocolate agar plate after 18-h incubation at 35°C in 5% CO_2_ via MagAttract HMW DNA kit (Qiagen, Toronto, ON, Canada). Nucleic acid was confirmed to be of high molecular weight (peak concentration value > 60 kb) using a Genomic DNA Screen Tape on a 4200 TapeStation System (Agilent, Santa Clara, CA, USA). About 400 ng of the bacterial high molecular weight DNA was used for a sequencing library preparation with the Rapid Barcoding Sequencing gDNA kit as per the manufacturer’s protocol (SQK-RBK004, Oxford Nanopore Technologies, Oxford, United Kingdom). The prepared library was loaded on an FLO-PRO002 sequencing flow cell and sequenced on a PromethION P2Solo device connected to GridION X5 machine running on MinKNOW 23.11.3 software with real-time high accuracy basecalling. For *de novo* bacterial genome assembly, 12,000 reads/58.9 M bases (N50: 13,530, N75: 6,037, and N90: 2,415) and representing an estimated 27× coverage of the expected genome size of 2.18 Mb (5) were processed with the Dragonflye pipeline (v1.1.2)(6) with one round of medaka polishing (v1.11.3; using model r941_prom_hac_g507) (7), and one round of racon polisher (v.1.5.0) (8). The assembly resulted in a single circular 2,182,197 bp contig representing 20× coverage. Taxonomic classification of the assembled genome was performed using Kraken2 (minikraken reference database) (9) to confirm the isolate was *H.somni*. The assembly was annotated using PGAP (10) identifying 2,118 genes and a GC content of 37.3%. ABRicate (11) using the default database (12) identified the presence of tet(H), aadA3, sul2, aph(3″)-Ib, aph (6)-Id, and aph(3′)-Ia antimicrobial genes within the genome.

**TABLE 1:** Antibiotic susceptibility test results for *Histophilus somni* PDS1537697-81-1^*a*^

Antibiotic	Minimum inhibitory concentration (µg/mL)	Interpretation
Ampicillin	0.3	R (≥0.25)
Ceftioufur	0.3	S (≤2)
Chlortetracycline	0.5	NI
Clindamycin	1.0	NI
Danofloxacin	0.1	NI
Enrofloxacin	0.1	S (≤0.25)
Florfenicol	0.3	S (≤2)
Gentamicin	16.0	NI
Neomycin	32.0	NI
Oxytetracycline	4.0	NI
Penicillin	0.1	S (≤0.1)
Spectinomycin	64.0	I (=64)
Sulfadimethoxime	256.0	NI
Tiamulin	2.0	NI
Tilmicosin	8.0	NI
Trimethoprim/sulfa	2.0	NI
Tulathromycin	16.0	S (≤16)
Tylosin	8.0	NI

^*a*^ Interpretation of breakpoints was done using CLSI VET01S-ED7:2024 Table 2J for antibiotics which have a clinically established breakpoint. Interpretation of breakpoints is reported as follows: S = susceptible, I = intermediate, R = resistant, and NI = no clinical interpretation criteria available.

## Data Availability

Data used to assemble the genome have been deposited in the SRA as the BioSample SAMN39905134. SRA data have been released and can be accessed as accession SRX23586881. Genome sequence has been released and is accessible through accession CP145800. Request for a bacterial stab of PDS1537697-81-1 can be made by contacting Prairie Diagnostic Services Inc., 52 Campus Drive, Saskatoon, Saskatchewan S7N 5B4, Canada, phone 1-306-966-7316, pds.info@usask.ca.

## References

[1] Shirbroun RM. 2020. Histophilus somni: antigenic and genomic changes relevant to bovine respiratory disease. Vet Clin North Am Food Anim Pract 36:279–295. doi:10.1016/j.cvfa.2020.02.003 32327251

[2] Besser J, Carleton HA, Gerner-Smidt P, Lindsey RL, Trees E. 2018. Next-generation sequencing technologies and their application to the study and control of bacterial infections. Clin Microbiol Infect 24:335–341. doi:10.1016/j.cmi.2017.10.013 29074157 PMC5857210

[3] Erickson NEN, Ngeleka MG, Lubbers BV, Trokhymchuk A. 2017 Changes in the rates of field isolation and antimicrobial susceptibility of bacterial pathogens collected from fallplaced feedlot steers between arrival at the feedlot and 90 to 120 days on feed. Bov pract 2017:165–173. doi:10.21423/bovine-vol51no2p165-173

[4] Wennekamp TR, Waldner CL, Windeyer MC, Larson K, Trokhymchuk A, Campbell JR. 2022. Antimicrobial resistance in bovine respiratory disease: auction market- and ranch-raised calves. Can Vet J 63:47–54. 34975167 PMC8682930

[5] Annotation of Histophilus somni USDA-ARS-USMARC-63369. 2022 Available from: https://www.ncbi.nlm.nih.gov/nuccore/NZ_CP01

[6] Petit RAI. 2024. Dragonflye: assemble bacterial isolate genomes from nanopore reads. Github2021. https://github.com/rpetit3/dragonflye.

[7] Oxford Nanopore Technologies. 2018. Sequence correction provided by ONT research

[8] Vaser R, Sović I, Nagarajan N, Šikić M. 2017. Fast and accurate de novo genome assembly from long uncorrected reads. Genome Res 27:737–746. doi:10.1101/gr.214270.116 28100585 PMC5411768

[9] Wood DE, Lu J, Langmead B. 2019. Improved metagenomic analysis with Kraken 2. Genome Biol 20:257. doi:10.1186/s13059-019-1891-0 31779668 PMC6883579

[10] Tatusova T, DiCuccio M, Badretdin A, Chetvernin V, Nawrocki EP, Zaslavsky L, Lomsadze A, Pruitt KD, Borodovsky M, Ostell J. 2016. NCBI Prokaryotic genome annotation pipeline. Nucleic Acids Res 44:6614–6624. doi:10.1093/nar/gkw569 27342282 PMC5001611

[11] Seemann T. 2018. Abricate. V1.0.0 Ed. Github2020. https://github.com/tseemann/abricate.

[12] Bacterial antimicrobial resistance reference gene database National library of medicine. 2024. Available from: https://www.ncbi.nlm.nih.gov/bioproject/PRJNA313047

